# Rules as to Behaviour during Sickness

**Published:** 1916-01-29

**Authors:** 


### NATIONAL INSURANCE ACT DEVELOPMENTS.

### Rules as to Behaviour during Sickness.

We had an opportunity, quite recently, of discussing National Insurance matters with a high official of an important group of approved societies, and were pleased to learn that the experience of those societies in regard to sickness claims during the past year had shown a marked improvement. We endeavoured to ascertain if there were any outstanding causes for this improvement, and were informed that several reasons might be given. In the first place the societies had paid greater attention than in the past to the supervision of claims. This supervision had been rendered possible by the proper organisation of sickness visitors, whose duties consist in visiting members in receipt of sickness benefit not only for the purpose of seeing that each case is in every sense *bona fide*, but also to see that as far as possible the appropriate steps are being taken to restore the member to health. In addition to this cause our informant also laid stress on the absence of unemployment, or the reduction in unemployment, and the higher wages that are now being earned, especially by women. These causes are manifestly of considerable importance, and while the latter should have operated to the advantage of all societies, the special advantage arising from the organisation of sickness visitors can only apply to the societies which have had the foresight to adopt such measures for the benefit not only of those members who are on the funds, but also indirectly for the benefit of the members as a whole.

We mention this matter because the Insurance Commissioners have recently issued a circular to approved societies (Circular A.S. 177) in which they call attention to the proposed alterations which are to be made by societies in their rules regarding behaviour during illness. Every society was bound under the provisions of the Act of 1911 to incorporate in its rules regulations as to the conduct of its members during illness, and these regulations had to be made in the form prescribed by the Commissioners. The Commissioners now state in their circular that, as the result of experience, it has been found that the form of rule at present prescribed does not make provision for certain points of importance, and they accordingly propose to make regulations prescribing a new form of rule.

### The New Form of Rule.

The proposed new form of rule is set out in the schedule to the proposed regulations which accompanied the circular, and is in the following terms, namely:—

"(1) A member who is incapable of work, and is or may become entitled to sickness or disablement benefit in respect of the incapacity:—(*a*) shall obey the instructions of the doctor in attendance, and shall answer any reasonable inquiries by the society as to the instructions given by the doctor; (*b*) shall not without the permission of the society, or some person duly authorised by them in that behalf, be absent from home between the hours of (*e.g.* 5) P.M. and (*e.g.* 9) A.M. from October 1 to March 31, nor between the hours of (*e.g.* 8) P.M. and (*e.g.* 8) A.M. from April 1 to September 30, and shall not be absent at any time without leaving word where he may be found; (*c*) shall not leave the . . . (town or area) without the consent of . . . (authority to be specified), but such consent shall not be unreasonably withheld; (*d*) shall not be guilty of conduct which is likely to retard his recovery; (*e*) shall not do any remunerative work; (*f*) shall not do any kind of heavy work, domestic or otherwise, such as . . . (*e.g.* baking, scrubbing, laundry-work, digging, grate-cleaning, window-cleaning, etc.), unless it be undertaken as a definite part of the member's treatment in an institution or in accordance with the rules of the institution; and (*g*) any further prohibition which the society may desire to insert.

"(2) The penalty for breach of this rule shall be a fine not exceeding 10s., or on a second time or later occasion 20s., or suspension from sickness and disablement benefits. Such suspension shall run from the date on which notice of the alleged breach of rule is sent to the member, or from such other date as the committee of management may determine, not being earlier than the commission of the alleged offence, and not being more than one week before the date on which notice is sent. The period of suspension shall be such as the committee of management determine, but shall not in any case exceed one year.

"(3) On receipt of a notice of illness from a member, the society shall furnish him with a copy of this rule at the first opportunity, and in any case not later than the date of the first payment of benefit."

### Points to be Noted.

There are several points in these new rules which are worthy of notice. In the first place it will be seen that the rules are to apply not only to those members who are actually receiving benefit, but also to those who *may become* entitled to benefit.

This is really more important than would appear at first sight, as it implies that the rules must have been obeyed during the first three days of illness, for which sickness benefit is not payable; and in the case of persons who pay a reduced rate of contribution in consequence of the relinquishment of right to benefit for the first six weeks of sickness (Section 47) it means that the rules must have been obeyed for that period of six weeks.

Again, the words in the latter half of (1) (*a*), which make it necessary for a member to answer reasonable inquiries as to the instructions given by the doctor, are new. It is evident that the intention of the Commissioners is to strengthen the hands of the societies, in order that the sickness visitors may be able to see that the member is following the doctor's instructions, and thus hastening the period of his recovery.

The headings (*b*), (*c*) and (*d*) in the new rule are substantially the same as in the existing rules, but they are rather more explicit, and are designed to hasten the recovery of the patient. It would hardly be possible for the average insured person to submit to the restrictions that are here imposed after he has regained his normal health, and although the restrictions are not made specifically from this point of view, it is probable that when they become fully known they will operate to shorten the duration of sickness claims.

The restrictions under headings (*e*) and (*f*) are quite new. They are in effect an attempt to define more exactly the meaning of the ambiguous expression "incapable of work." The precise work to be excluded under the description of heavy work is to be inserted by the individual society, and the character of the society will determine the words that are to be employed. It is clear from the suggestions that have been inserted by the Commissioners that an attempt is to be made, by the more stringent application of the rules regarding conduct during sickness, to reduce the heavy rate of sickness claim among married women.

### The Penalties.

In order that the rules may be made effective it is, of course, essential to provide appropriate penalties for their infringement. The penalty may take either the form of a specific money fine, or of a suspension from benefit. Suspension from benefit, if imposed, must not be for a period exceeding a year, and in their circular the Commissioners draw special attention to the distinction that is to be drawn between the suspension of a member who is actually entitled to benefit immediately, and of a member who is not so entitled. They suggest that the matter should be looked at from the point of view of the money equivalent.

It is supposed that every insured person has a copy of the rules of the approved society of which he is a member; if he has not, he is at least entitled to a copy—but this does not mean that every insured person is familiar with such rules. It is therefore a salutary provision of the proposed new rules that each member shall be furnished with a copy of the rule regarding his conduct during illness not later than the payment of the first instalment of benefit. The members will thus have no cause to complain that any breach of the rules was made in ignorance. Moreover the Commissioners impress upon the societies the importance of adopting a clear and unambiguous manner in inflicting any penalty upon a member. They also indicate the necessity of giving full opportunity to a member to state his case, when any complaint has been made as to his conduct, before the committee of management decides to inflict a penalty.

The effectiveness of the new rules will necessarily depend in large measure upon the spirit in which they are carried out. They will require a certain amount of firmness on the part of the officials and representatives of the societies, and it is hardly to be expected that they will not cause some irritation at the outset among those members who have not been used to strict observance of the existing rules. At the same time it would appear that the new rules are a great improvement upon the existing ones, and in the long run the members should be educated to see that they are framed in the best interests of those who are ill, just as much as they are necessary to protect the members as a whole from claims by members who strictly have no right to benefit. If properly carried out, the effect of the new rules should be very similar to that indicated at the beginning of this article—namely, that by efficient supervision of claims the rate of sickness should be materially reduced.

